# B-box transcription factor 28 regulates flowering by interacting with constans

**DOI:** 10.1038/s41598-020-74445-7

**Published:** 2020-10-20

**Authors:** Yin Liu, Guang Lin, Chunmei Yin, Yuda Fang

**Affiliations:** 1 Joint Center for Single Cell Biology, School of Agriculture and Biology, Shanghai Jiao Tong University, Shanghai, 200240 China; 2 National Key Laboratory of Plant Molecular Genetics, CAS Center for Excellence in Molecular Plant Sciences, Institute of Plant Physiology and Ecology, Chinese Academy of Sciences, University of Chinese Academy of Sciences, Shanghai, 200032 China

**Keywords:** Cell biology, Developmental biology, Plant sciences

## Abstract

B-box transcription factors (BBXs) are important regulators of flowering, photomorphogenesis, shade-avoidance, abiotic and biotic stresses and plant hormonal pathways. In *Arabidopsis*, 32 BBX proteins have been identified and classified into five groups based on their structural domains. Little is known about the fifth group members (BBX26–BBX32) and the detailed molecular mechanisms relevant to their functions. Here we identified B-box transcription factor 28 (BBX28) that interacts with Constans (CO), a transcriptional activator of *Flowering Locus T* (*FT*). Overexpressing *BBX28* leads to late flowering with dramatically decreased *FT* transcription, and *bbx28* deficient mutant displays a weak early flowering phenotype under long days (LD), indicating that BBX28 plays a negative and redundant role in flowering under LD. Additionally, the interaction between BBX28 and CO decreases the recruitment of CO to *FT* locus without affecting the transcriptional activation activity of CO. Moreover, the N-terminal cysteines, especially those within the B-box domain, are indispensable for the heterodimerization between BBX28 and CO and activation of CO on *FT* transcription. Genetic evidences show that the later flowering caused by *BBX28* overexpression is compromised by *CO* ectopic expression. Collectively, these results supported that BBX28 functions with CO and *FT* to negatively regulate *Arabidopsis* flowering, in which the N-terminal conserved cysteines of BBX28 might play a central role.

## Introduction

Flowering is an essential biological process for the transition from vegetative to reproductive stage in responses to internal and external signals^1^. Five main pathways have been identified in flowering regulation, including vernalization, autonomous, photoperiodic, gibberellin and age pathways^2^. Ambient temperature, sugar, histone modifications, small RNAs and chromatin loops also play roles in flowering regulation^2,3^. Constans (CO)-*Flowering Locus T* (*FT*) is the central module in photoperiodic pathway^4^. CO, the first member of B-box transcription factors (BBX1), binds to the *CORE1* and *CORE2* motifs of *FT* promoter to activate its expression^5^. FT interacts with bZIP transcription factor FD to promote the expressions of floral identity genes and induce floral meristem formation^6^.

The transcriptional and post-translational regulations of CO ensure the precise protein level of CO during the day-night and the correct time of *FT* expression under long day (LD) condition to induce photoperiodic flowering^4,7^. At the post-translational level, the stability and activity of CO protein are regulated by multiple CO-interacting proteins^4,7^. Two RING-finger E3 ubiquitin ligases Constitutive Photomorphogenic 1 (COP1) and High Expression of Osmotically Responsive Genes 1 (HOS1) mediate the degradation of CO at night and in the morning respectively^8,9^. Photoreceptors Phytochrome B (PHYB) and Zeitlupe (ZTL) destabilize CO in the morning while Phytochrome A (PHYA), Cryptochromes (CRYs) and Flavin-Binding, Kelch Repeat F-box1 (FKF1) stabilize CO in the afternoon under LD^10–13^. The interaction between Nucleoporin 96 (Nup 96) and HOS1 and their mutual stabilization form a novel repressive module to gate CO protein level in *Arabidopsis* under LD^14^. GIGANTEA (GI) plays a pivotal regulation on the timing stabilization of CO by altering FKF1-ZTL interaction^15^. Other CO-interacting proteins, including BBX19, microProtein 1a (miP1a, or BBX30) and miP1b (or BBX31), Target of EAT1 (TOE1), TOE2, Nuclear Factor Y (NF-Y) transcription factors, Della protein RGA, Botrytis Susceptible 1 Interactor (BOI), Vascular Plant One-Zinc Finger 1 (VOZ1), VOZ2 and immunophilin FKBP12, regulate flowering by affecting CO transcriptional activity or DNA binding ability^16–23^. Pseudo Response Regulator (PRR) proteins regulate both *CO* transcription and CO protein stabilization, increasing the recruitment of CO to *FT* promoter^24^. CRY2-interacting bHLH1(CIB1), CO and CRY2 form a complex through physical interaction between CIB1 and CO to regulate *FT* expression and CRY2-dependent flowering^25^. Long Hypocotyl in FAR-RED 1 (HFR1) interacts with CO and Phytochrome-Interacting Factor 7 (PIF7) to repress early flowering under the shaded environment^26^.

B-box transcription factors are a class of zinc finger binding proteins, which contain one or two B-box domains in the N-terminus and sometimes a CCT (CONTANS, CO-like and TOC1) domain in the C-terminus^27,28^. Based on the numbers of B-box domains and the existence of CCT domain, the 32 B-box proteins in *Arabidopsis* are classified into five groups. BBX26-BBX32 belong to the fifth group, which contains only one B-box domain in the N-terminus but no CCT domain in the C-terminus^27^. BBX1 (CO) and many other B-box proteins were known to regulate flowering in CO-dependent or independent manners^27^. The suppression of flowering by BBX4 (COL3) depends on the BBX32-BBX4 interaction which contributes to the targeting of BBX4 to *FT* promoter to inhibit *FT* expression^29,30^. Overexpressing BBX6 causes early flowering by increasing the transcripts of *FT* and *SUPPRESSOR OF OVEREXPRESSION OF CO 1* (*SOC1)* but not *CO* under short day (SD) condition^31^. Overexpression of BBX7 delays flowering under LD through decreasing *CO*, *FT* and *SOC1* mRNA levels^32^. BBX19 decreases *FT* transcript level and represses flowering under LD^16^. BBX24 (SALT TOLERANCE, STO) promotes flowering under both SD and LD conditions by reducing *FLOWERING LOCUS C* (*FLC*), *FT* and *SOC1* expressions^33^. BBX30 and BBX31 interact with CO and TOPLESS (TPL) to suppress *FT* expression^19^. BBX32 interacts with EMBRYONIC FLOWER 1 (EMF1) to regulate flowering^34^. Heading date 1(Hd1), OsBBX5, OsBBX14, OsBBX27 or OsCOL9 in rice play positive or negative roles in flowering under LD or SD conditions^35–39^. *Chrysanthemum* BBX8 and BBX13 were reported to regulate flowering^40,41^. At the cellular level, CO and BBX4 colocalize with Constitutive Photomorphogenic 1 (COP1) in photobodies through interaction with COP1^9,30^.

In this study, we identified a new CO-interacting protein BBX28 and revealed that BBX28 functions through its N-terminal cysteine-mediated heterodimerization with CO to weaken the role of CO in transcriptional regulation of *FT* to negatively regulate *Arabidopsis* flowering.

## Results

### BBX28 negatively regulates flowering

**Figure 1:**
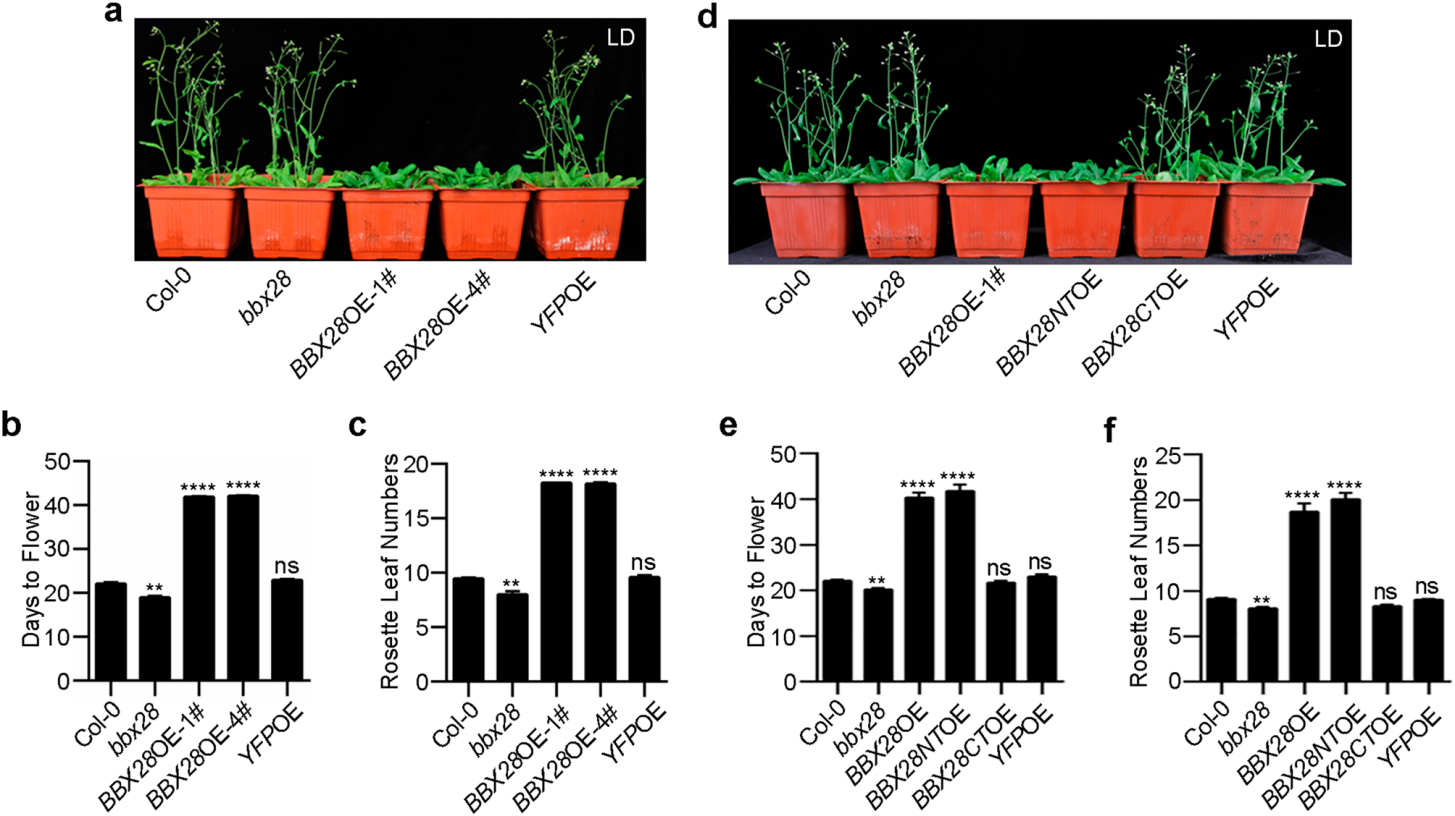
BBX28 negatively regulates flowering. (**a**) Visual phenotypes of 33-day-old Col-0, *bbx28*, and plants overexpressing *YFP* or *BBX28* under LD. *BBX28*OE: *35S-BBX28-YFP*/Col-0; *YFP*OE: *35S-YFP*/Col-0. Numerals with the pound sign represent independent lines. (**b**, **c**) Flowering time and rosette leaf numbers of genotypes in (**a**) (n = 4 biological replicates; plant number ≥ 9 in each replicate). (**d**) Visual phenotypes of 33-day-old Col-0, *bbx28*, and plants overexpressing *BBX28* or its truncations under LD. *BBX28NT*OE: *35S-BBX28NT-YFP*/Col-0; *BBX28CT*OE: *35S-BBX28CT-YFP*/Col-0. (**e**, **f**) Flowering time and rosette leaf numbers of genotypes in (**d**) (n = 5 biological replicates; plant number ≥ 9 in each replicate). Data are means ± SEM. Statistical significance was analyzed by student’s *t*-test; ****p < 0.0001, **p < 0.01; ns, not significant.

To uncover the biological functions of BBX28 in addition to its role in the regulation of photomorphogenesis^42^, we obtained a *bbx28* mutant with a T-DNA insertion in the first exon without a detectable *BBX28* transcript level (Fig. S1a,b), and two independent transgenic lines overexpressing *BBX28* fused to yellow fluorescent protein (*YFP*) under the control of a CaMV 35S promoter in wild type (Col-0) (*BBX28*OE, *35S-BBX28-YFP*/Col-0; lines 1# and 4#) (Fig. S1b), while Col-0 and plants overexpressing *YFP* (*YFP*OE, *35S-YFP*/Col-0) were used as controls. When these plants were grown under LD, *bbx28* mutant flowered two days earlier with one rosette leaf less than Col-0, while *BBX28*OE lines showed severely late flowering phenotype, with about twenty days later and ten more rosette leaves than Col-0 (Fig. 1a–c). As the two independent *BBX28*-overexpressing lines display similar phenotypes (Fig. 1a–c), we used *BBX28*OE-1# for further analysis.

We crossed *BBX28*OE with *bbx28* mutant to obtain *BBX28*OE/*bbx28* plants. We found that *BBX28*OE/*bbx28* plants flowered similarly to *BBX28*OE plants (Fig. S1c–e). The weak phenotype of *bbx28* mutant might be due to the functional redundancies of *Arabidopsis* group V BBX proteins in flowering regulation (Fig. S1f).

To map the functional domains of BBX28 in flowering repression, we constructed N-terminal (BBX28NT, 1–112 aa) and C-terminal (BBX28CT, 113–223 aa) truncations of BBX28 based on their protein domains predicted by SMART (https://smart.embl-heidelberg.de/), which revealed a B-box domain (1–46 aa) in the N-terminus and two low-complexity regions in the C-terminus of BBX28 (Fig. S1g). We generated transgenic plants overexpressing *BBX28NT* or *BBX28CT* in Col-0 (*BBX28NT*OE, *35S-BBX28NT-YFP*/Col-0; *BBX28CT*OE*, 35S-BBX28CT-YFP*/Col-0) (Fig. S1h). We found that *BBX28NT*OE lines showed severely late flowering, with about twenty days later and ten more rosette leaves than Col-0. In contrast, *BBX28CT*OE and *YFP*OE lines displayed similar flowering phenotypes to Col-0 (Fig. 1d–f), suggesting a specific role of BBX28NT in the flowering regulation.

### BBX28 decreases *FT* transcription

**Figure 2:**
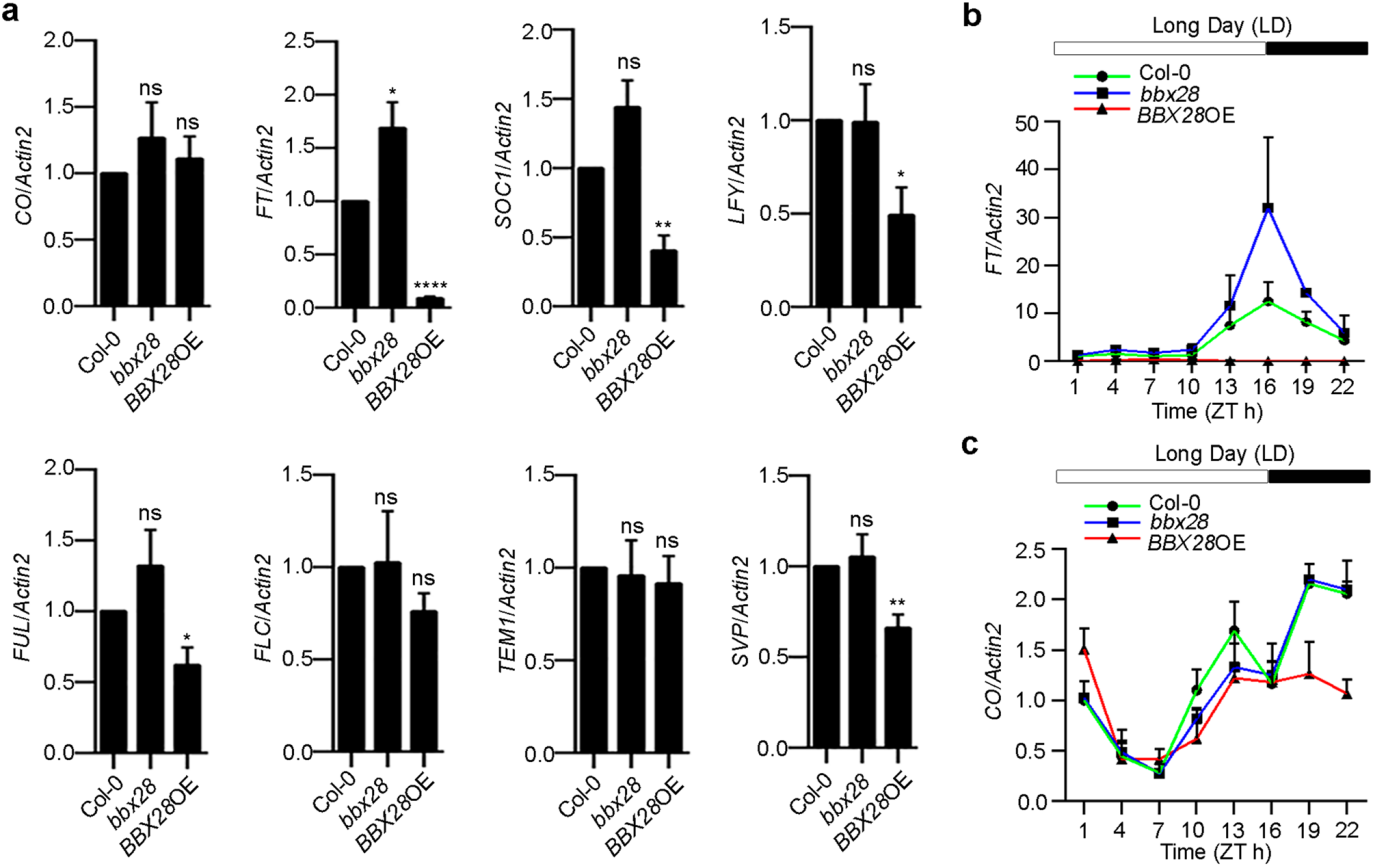
BBX28 decreases *FT* transcription. (**a**) Transcript levels of flowering-related genes in 7-day-old LD-grown Col-0, *bbx28* and *BBX28*OE (*35S-BBX28-YFP*/Col-0) *Arabidopsis* plants. Seedlings were collected at zeitgeber time (ZT) 4 for RNA extraction and qRT-PCR assays (n = 6 biological replicates). (**b**, **c**) Diurnal levels of *FT* transcript (**b**) or *CO* transcript (**c**) in Col-0, *bbx28* and *BBX28*OE plants (n = 3 biological replicates). *Actin2* was used for data normalization in qRT-PCR assays. Data are means ± SEM. Statistical significance was analyzed by student’s *t*-test; ****p < 0.0001, **p < 0.01, *p < 0.05; ns, not significant.

To reveal genes involved in the negative role of BBX28 in regulating flowering, we tested the transcript levels of key genes involved in photoperiodic flowering*,* floral identity and *FT* transcription, including *CO*, *FT*, *SOC1, LEAFY* (*LFY*), *FRUITFUL* (*FUL*), *TEMPRANILLO 1* (*TEM1*), *FLC* and *SHOT VEGETATIVE PHASE* (*SVP*)^16^, in 7-day-old LD-grown Col-0, *bbx28* and *BBX28*OE plants. Quantitative real-time PCR (qRT-PCR) of seedlings collected at zeitgeber time (ZT) 4 showed that *FT* transcript level reduced most significantly in *BBX28*OE, followed by *SOC1*, *LFY*, *FUL* and *SVP,* while *CO*, *TEM1* and *FLC* transcript levels remained unchanged (Fig. 2a).

As the expressions of *CO* and *FT* show rhythmic patterns during 24 h of a day in flowering pathway^4^, we tested whether the diurnal expression profiles of *FT* and *CO* are affected by BBX28. To this end, 7-day-old Col-0, *bbx28* and *BBX28*OE seedlings grown under LD were harvested with an interval of three hours from dawn up to a 24 h-period. We found that *FT* transcript level was greatly decreased by BBX28 with more dramatically in late afternoon and dark (Fig. 2b). Interestingly, *CO* transcription was not affected by BBX28 during the day but decreased about half in *BBX28*OE plants after dusk (Fig. 2c).

### BBX28 interacts with CO through its N-terminus

**Figure 3:**
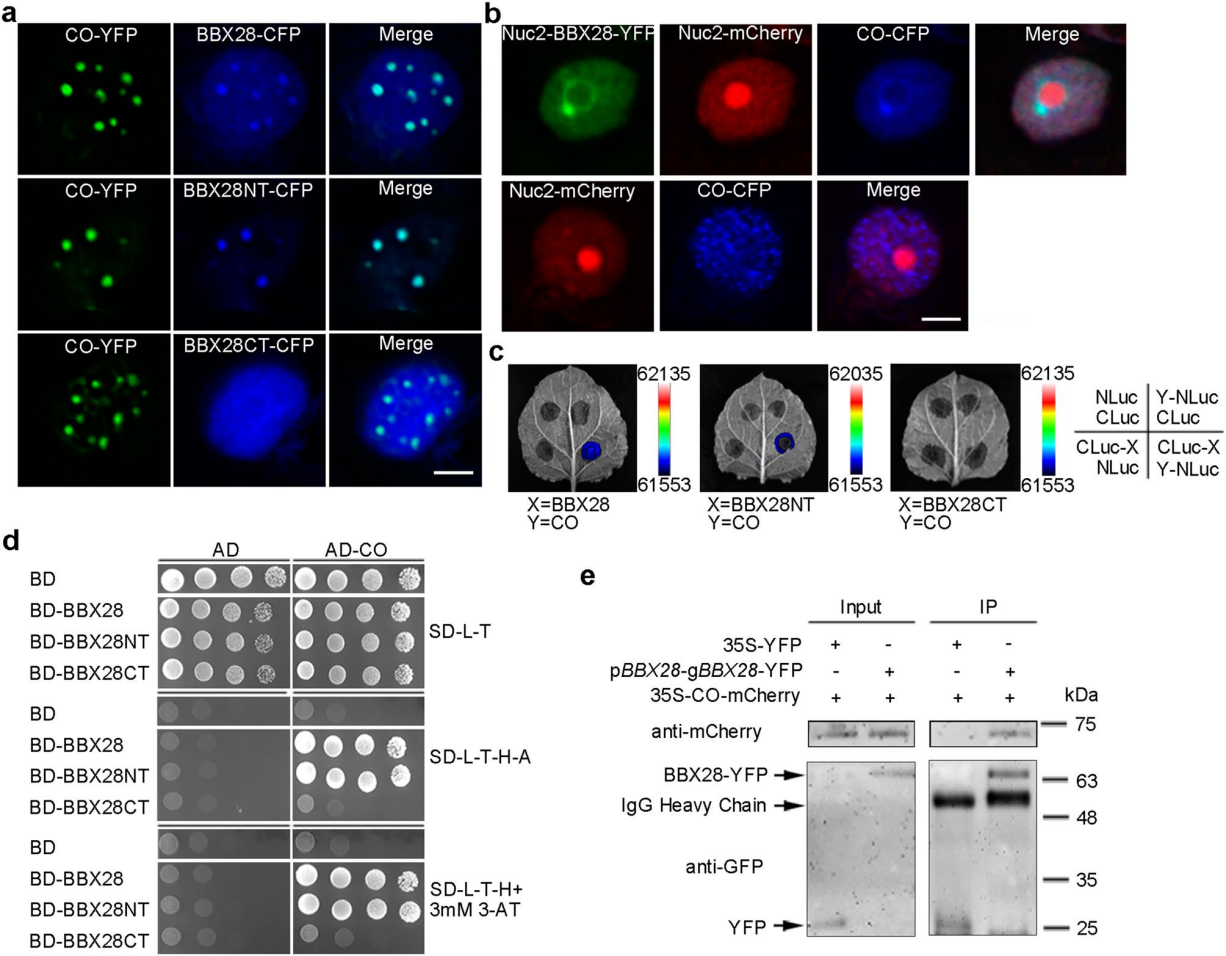
BBX28 interacts with CO through its N-terminus. (**a**) Co-localizations of CO and BBX28 in tabacco leaves. Bar = 5 μm. (**b**) NoTS assays of the interaction between BBX28 and CO in tobacco. The nucleolus was labeled by Nuc2-mCherry. Bar = 5 μm. (**c**) LUC complementation imaging assays of CO-BBX28 interaction in tobacco. NLuc/CLuc, Y-NLuc/CLuc and NLuc/CLuc-X acted as negative controls. (**d**) Yeast two-hybrids of BBX28 or BBX28 truncations with CO. Cells were grown on selective plates for interaction assays. (**e**) Co-IP assay of BBX28-CO interaction. Tobacco leaves co-expressing CO-mCherry and YFP were used as the negative controls. Proteins were detected by western blots with anti-GFP or anti-mCherry antibodies. Molecular weight standards are indicated. The full-length blots are presented in Fig. S2a.

Since *FT* is the direct target of CO, the reduction of *FT* transcript level by BBX28 encouraged us to test whether BBX28 interacts with CO. Co-localization analyses revealed that BBX28 and BBX28NT but not BBX28CT co-localized with CO in photobodies (Fig. 3a). We performed NoTS assay^43^ to test the interaction between BBX28 and CO in vivo. We found that CO was successfully recruited to the periphery of nucleolus by Nuc2-BBX28 but not Nuc2-mCherry (Fig. 3b), indicating that BBX28 interacts with CO in nuclei in vivo. LUC complementation imaging and yeast two-hybrid interaction assays also supported the interaction between BBX28 and CO through BBX28NT (Fig. 3c,d). The in vivo interaction between BBX28 and CO was further confirmed by Co-IP as CO was detected in BBX28-YFP-immunoprecipitated samples but not in the control (Figs. 3e; S2a). To study whether the B-box domain within N-terminus of BBX28 mediates its interaction with CO, BBX28 B-box (1–46aa) and BBX28NT with a deletion of the B-box domain (BBX28NTΔB-box, 47–112aa) were constructed (Fig. S2b). Yeast two-hybrids showed that the B-box domain but not BBX28NTΔB-box interacts with CO (Fig. S2c), suggesting a central role of B-box domain in mediating the interaction between BBX28 and CO.

### BBX28 inhibits CO targeting to *FT* locus without affecting the transcriptional activation activity of CO

**Figure 4:**
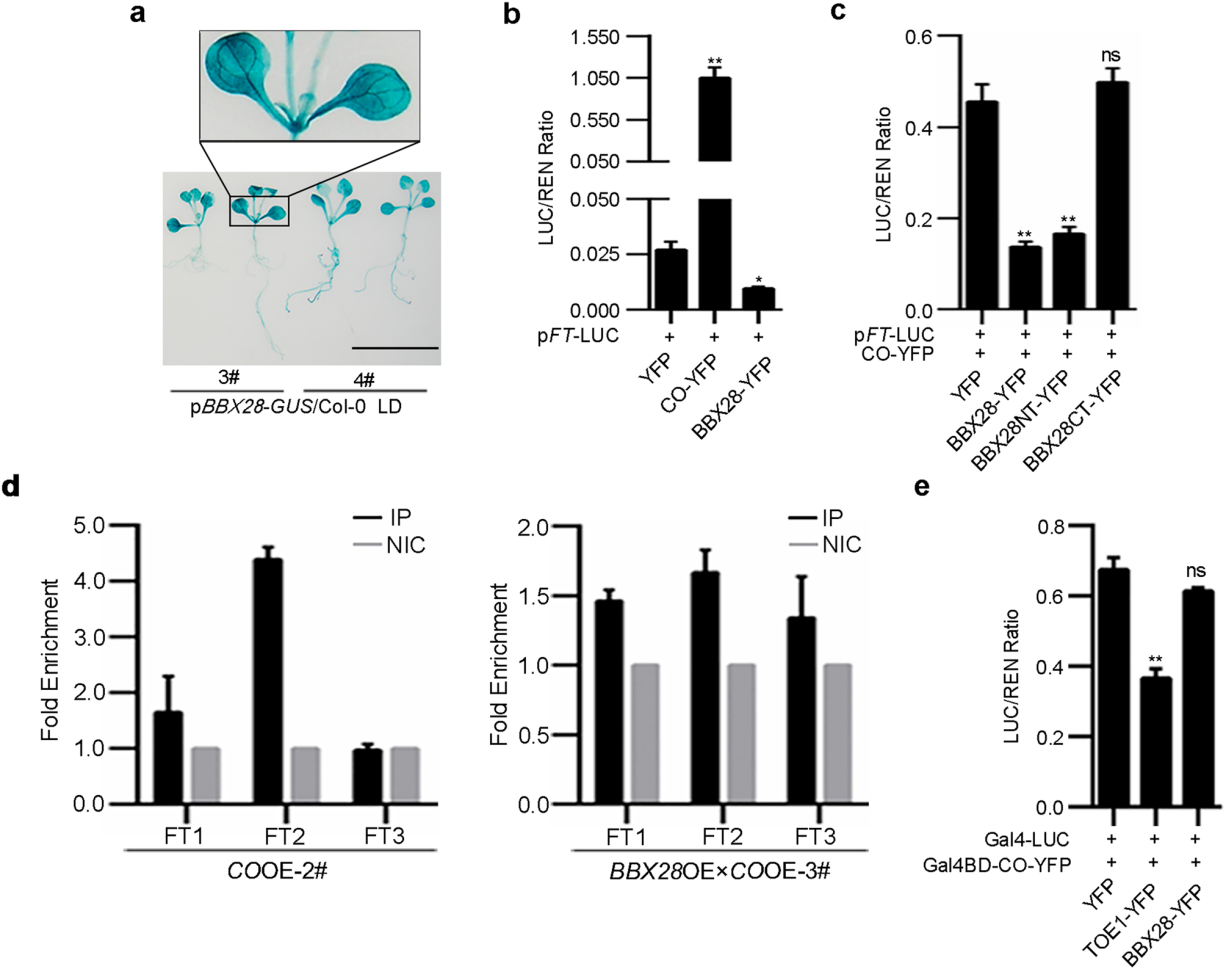
BBX28 inhibits CO targeting to *FT* locus without affecting the transcriptional activation activity of CO. (**a**) Histochemical GUS staining of p*BBX28-GUS*/Col-0 seedlings. Numerals with the pound sign represent independent lines. Bar = 1 cm. (**b**) Dual-LUC assays show the effects of CO and BBX28 on *FT* transcription. (**c**) Dual-LUC assays show the effects of co-expressing CO and BBX28 or BBX28 truncations on *FT* transcription. (**d**) ChIP-qPCRs show the effects of BBX28 on CO targeting to *FT* locus. 12-day-old LD-grown seedlings were harvested at ZT12 for ChIP-qPCR assays using an anti-GFP antibody. IP (GFP antibody): immunoprecipitation; NIC (no GFP antibody): nonimmune control. ChIP results were presented by the fold enrichment. GFP-IP signal relative to corresponding NIC signal was normalized by FT4. Similar results were observed in two independent biological replicates. *CO*OE: *35S-CO-YFP*/Col-0; *BBX28*OE: *35S-BBX28-3* × *FLAG-mCherry*/Col-0. (**e**) The transcriptional activity assays show the effects of BBX28 on the transcriptional activation activity of CO. YFP served as a negative control. TOE1 served as a positive control. Data are means ± SEM. In (**b**), n = 4 biological replicates. In (**c**) and (**e**), n = 3 biological replicates. Statistical significance was analyzed by student’s *t*-test; **p < 0.01, *p < 0.05; ns, not significant.

To investigate the functional links among BBX28, CO and *FT*, we first compared the expression patterns among *BBX28*, *CO* and *FT.* Transgenic plants expressing *GUS* driven by the *BBX28* promoter in wild type (p*BBX28-GUS*/Col-0) were generated and GUS staining showed that the expression pattern of *BBX28* is similar to those of *CO* and *FT*^44,45^, with higher levels in vascular tissues (Fig. 4a). Dual-LUC assays (Fig. S3a) showed that the *FT* expression level was increased by CO but decreased by BBX28 (Fig. 4b), indicating a negative effect of BBX28 on *FT* transcription. Since CO activates *FT* transcription and BBX28 interacts with CO (Figs. 3 and 4b), we co-expressed BBX28 and CO and performed Dual-LUC assays to test the effect of coexpressing *BBX28* and *CO* on *FT* expression. The results indicated that the *FT* transcript level was reduced upon co-expressing BBX28 and CO (Fig. 4b,c). Moreover, BBX28NT but not BBX28CT displayed a similar effect on *FT* transcription to that of full-length BBX28 upon co-expressing with CO (Fig. 4c).

It was known that CO binds to *FT* promoter to activate its expression^5,17^. We asked whether BBX28 affects the DNA binding ability of CO to *FT* locus. Plants overexpressing *CO* (*CO*OE; *35S-CO-YFP*/Col-0) were crossed with *BBX28*OE line (*BBX28*OE; *35S-BBX28-3* × *FLAG-mCherry*/Col-0) to obtain *CO* and *BBX28* co-expressing lines (*BBX28*OE × *CO*OE). The line 1#, 3#, and 5# of *BBX28*OE × *CO*OE were generated by crossing *BBX28*OE-2# with line 1#, 2#, and 6# of *CO*OE respectively (Fig. S3b,c). *CO*OE-2# and *BBX28*OE × *CO*OE-3#, having a similar CO protein level which excluded the potential effects of different CO levels on our analysis (Fig. S3d), were selected for chromatin immunoprecipitation (ChIP)-qPCR assay. The level of CO recruited to the transcription start site of *FT* (*FT2* fragment) was much more than that to other fragments of *FT* in *CO*OE plants, exhibiting enrichment over four folds compared to the non-immune control (NIC) (Figs. 4d, S3e). When CO was co-expressed with BBX28, the level of CO targeted to *FT2* fragment decreased to less than two folds compared to NIC (Fig. 4d), indicating that BBX28 decreases the recruitment of CO to *FT* locus.

Next we tested whether BBX28 affects the transcriptional activation activity of CO (Fig. S3f). The expression of *LUC* (LUC/REN ratio) increased in Gal4BD-CO-YFP and Gal4BD-VP16-YFP (Fig. S3g), consistent with the previous report^5^. When Gal4BD-CO-YFP was co-expressed with BBX28 or TOE1, a positive control which was shown to repress the transcriptional activation of CO^18^, we found that the transcriptional activation activity of CO was not affected by BBX28 while inhibited obviously by TOE1 (Fig. 4e). Without Gal4BD-CO-YFP, BBX28 and TOE1 had no effects on the reporter *LUC* (Fig. S3h). Together, these results indicated that BBX28 does not affect the transcriptional activation activity of CO.

### BBX28 N-terminal cysteines mediate heterodimerization between BBX28 and CO and affect the activation of CO on *FT* transcription

**Figure 5:**
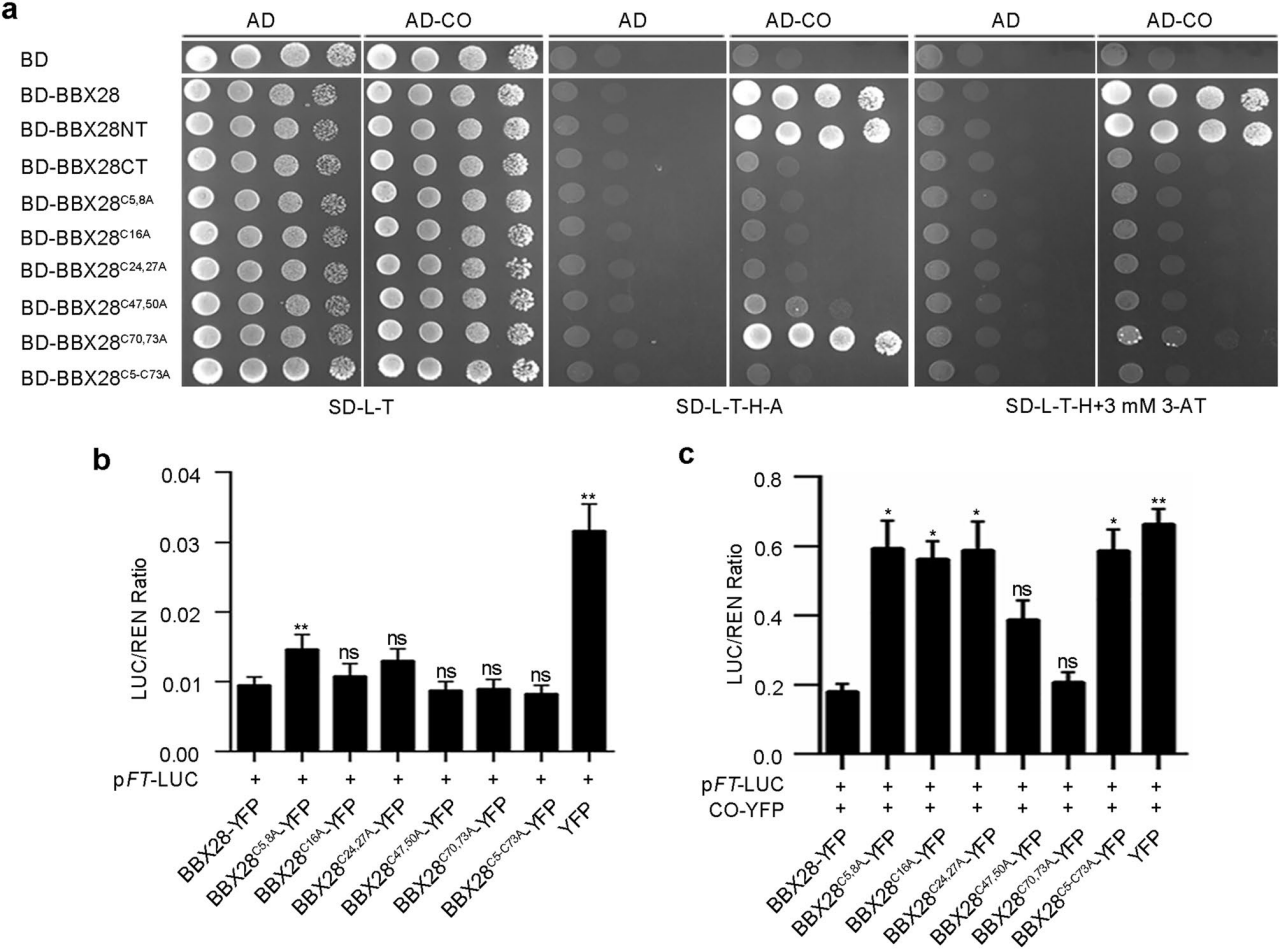
BBX28 N-terminal cysteines mediate heterodimerization between BBX28 and CO and affect the activation of CO on *FT* transcription. (**a**) Yeast two-hybrids between CO and BBX28, BBX28 truncations or BBX28 cysteine mutations. Cells were grown on selective plates for interaction assays. (**b**) Dual-LUC assays show the effects of BBX28 cysteine mutants on *FT* expression (n = 4 biological replicates). (**c**) Dual-LUC assays show the effects of co-expressing CO and BBX28 cysteine mutants on *FT* expression (n = 3 biological replicates). Data are means ± SEM. Statistical significance was analyzed by student’s *t*-test; **p < 0.01, *p < 0.05; ns, not significant.

As BBX28 interacts with CO through its N-terminus (Fig. 3), we then asked whether the conserved cysteines in N-terminal domain (Fig. S4a) play a role in BBX28-CO interaction. We found that mutations of BBX28 N-terminal cysteines greatly blocked BBX28-CO interaction with BBX28^C5,8A^, BBX28^C16A^ and BBX28^C24,27A^ displaying more obvious blocking effects, followed by BBX28^C47,50A^ and BBX28^C70,73A^ (Fig. 5a). Consistently, the co-localization signals in photobodies between CO and BBX28^C5,8A^ or BBX28^C16A^ decreased significantly, followed by BBX28^C24,27A^, BBX28^C47,50A^ and BBX28^C70,73A^ mutants. BBX28^C5–C73A^ with all nine conserved cysteines mutated diffused in the nucleus without photobodies formed when co-expressing with CO (Fig. S4b,c). These results indicated that the N-terminal cysteines are essential for BBX28-CO interaction and C5, C8, C16, C24 and C27 sites located in the B-box domain (1–46aa) are more important. As BBX28 and CO (BBX1) both belong to BBX proteins, we concluded that BBX28 N-terminal cysteines mediated its heterodimerzation with CO.

To test whether these cysteines affect *FT* transcription, Dual-LUC assays showed that BBX28 cysteine mutations did not change the effects of BBX28 on *FT* expression, except BBX28^C5,8A^ mutation with less than one-fold increase (Fig. 5b). When those mutations were co-expressed with CO, BBX28^C5,8A^, BBX28^C16A^, BBX28^C24,27A^ and BBX28^C5–73A^ mutations, which affect the interaction between BBX28 and CO (Fig. 5a; Fig. S4b,c), largely restored *FT* expression (Fig. 5c), supporting that these cysteines play an inhibitory role in the activation of CO on *FT* transcription, possibly by affecting the heterodimerization between BBX28 and CO.

### BBX28 genetically regulates flowering through CO and *FT*

**Figure 6:**
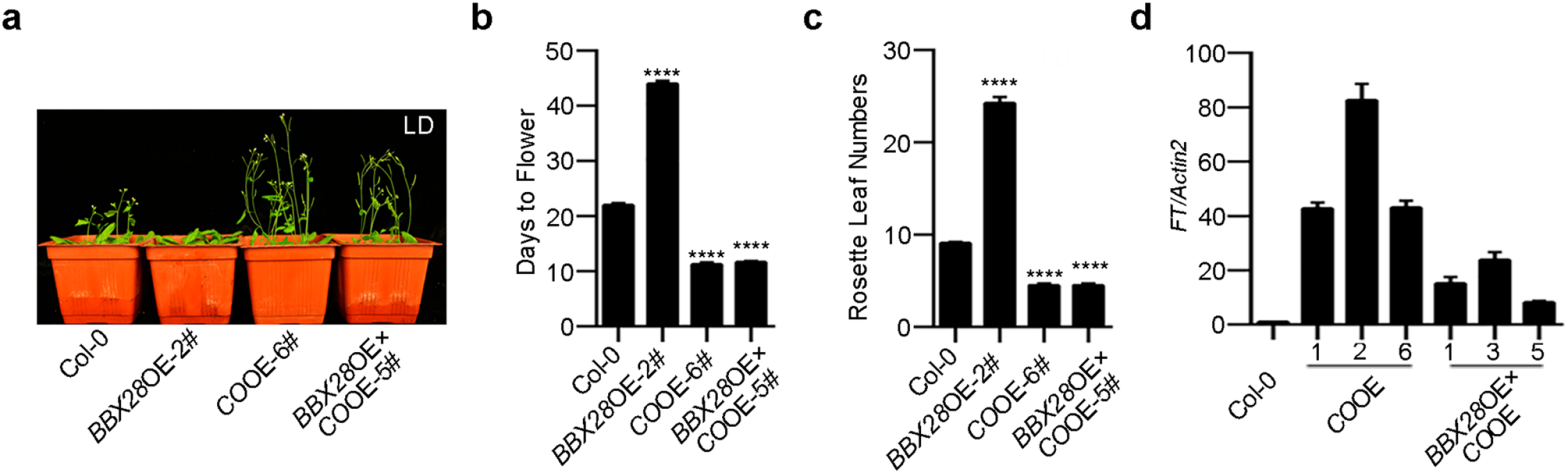
BBX28 genetically regulates flowering through CO and *FT*. (**a**) Visual phenotypes of 27-day-old Col-0, *BBX28*, *CO* overexpressing plants or *BBX28* and *CO* co-overexpressing plants under LD. *CO*OE: *35S-CO-YFP*/Col-0; *BBX28*OE: *35S-BBX28-3* × *FLAG-mCherry*/Col-0. (**b**, **c**) Flowering time and rosette leaf numbers of different genotypes in (**a**). (**d**) qRT-PCR analyses of *FT* expression in Col-0, *CO* overexpressing plants or *BBX28* and *CO* co-overexpressing plants. In (**b**, **c**), data are means ± SEM (n = 3 biological replicates; plant numbers ≥ 18 in each replicate). In (**d**), *Actin2* was used for data normalization. Data are mean ± SD (n = 3 technical replicates). Statistical significance was analyzed by student’s *t*-test; ****p < 0.0001.

To test the genetic relationships among *BBX28*, *CO* and *FT*, we first generated *bbx28co-9* double mutant by crossing *bbx28* with *co-9* mutant and analyzed their flowering phenotypes under LD. We found *bbx28co-9* double mutant delayed flowering similarly to *co-9* single mutant (Fig. S5a–c). As the early flowering phenotype of *bbx28* is weak (Fig. 1) while *co* mutant (*co-9*) displays severe late flowering (Fig. S5a–c), we then tested the genetic relationship between *BBX28* and *CO* through analyzing the flowering phenotypes of plants overexpressing *BBX28* (*BBX28*OE), *CO* (*CO*OE) or *BBX28* and *CO* (*BBX28*OE × *CO*OE). The line 1#, 3#, and 5# of *BBX28*OE × *CO*OE were generated by crossing *BBX28*OE-2# with line 1#, 2#, and 6# of *CO*OE respectively, and the transcript levels of *CO* and *BBX28* were confirmed by qRT-PCRs (Fig. S3b,c). We found that *BBX28*OE × *CO*OE-5# flowered early with about twelve days earlier and five rosette leaves less than Col-0, similar to *CO*OE-6# plants (Fig. 6a–c); *BBX28*OE × *CO*OE-1# flowered early with about eleven days earlier and four rosette leaves less than Col-0, similar to *CO*OE-1# plants (Fig. S5d–f); *BBX28*OE × *CO*OE-3# flowered early with about ten days earlier and four rosette leaves less than Col-0, similar to *CO*OE-2# plants (Fig. S5g–i), indicating the later flowering caused by *BBX28* overexpression was compromised by *CO* overexpression. *BBX28*OE × *CO*OE plants flowered no later than *CO*OE plants, which might be due to that the high levels of *CO* transcripts in both genotypes, resulting in a saturated level of *FT* transcript for promoting flowering. Moreover, *FT* transcripts were much less in *BBX28*OE × *CO*OE plants than that in *CO*OE plants (Fig. 6d), although the *CO* transcript and CO protein levels were comparable in *BBX28*OE × *CO*OE plants and corresponding *CO*OE plants (Fig. S3b,d), supporting a negative effect of BBX28 on CO function in vivo. In addition, *FT* mRNA level changed in accordance with *CO* transcript level in *BBX28*OE × *CO*OE plants (1#, 3# and 5#) (Figs. 6d, S3b). *BBX28*OE × *CO*OE-3# showed the highest *CO* transcript level, followed by *BBX28*OE × *CO*OE (1# and 5#) (Fig. S3b), accordantly, the *FT* transcript level displayed similar patterns to *CO*, with highest in *BBX28*OE × *CO*OE-3#, followed by *BBX28*OE × *CO*OE (1# and 5#) (Fig. 6d). Together, these results suggested that BBX28 delays flowering in a CO and *FT*-dependent manner.

## Discussion

The CO-FT module is the major determinant to regulate flowering in response to day length. In this study, we identified a new CO-interacting protein BBX28 which belongs to the Group V B-box transcription factors (BBX26–BBX32). We further dissected a specific role of N-terminal domain of BBX28 in the negative regulation of flowering.

In the light signaling pathways, it was shown that some components function through their different protein domains intelligently. The C-terminal domains of CRY1 and CRY2 (CCT1 and CCT2) interact with COP1 and mediate their signaling responses to light activation. The N-terminal domain of CRY1 (CNT1) mediates its constitutive dimerization, which is required for the light activation of CCT1 activity^46,47^. COP1 is a central switch for light signaling transduction. The nuclear localization signals (NLSs) of COP1 reside in its central core domain while the N-terminal portion acts as the major determinant for its cytoplasmic distribution. The coiled-coil domain of COP1 mediates its homodimerization and the C-terminal WD40 domain interact with multiple transcription factors or photoreceptors for protein degradation^48–50^. Our results showed that the N-terminus of BBX28 mediates its interaction with CO and affects the recruitment of CO to *FT* locus, resulting in reduction of *FT* transcription with delayed flowering under LD (Figs. 3, 4 and 6). The late flowering phenotype is relieved when BBX28 N-terminus is deleted (Figs. 1d–f, 4c), supporting specific roles of the regulatory protein domains in the light signaling transduction.

The protein interactions, such as BBX19-CO, BBX32-BBX4 and BBX21-HY5, indicated heterodimer formation among BBXs or between BBX and non-BBX proteins^16,27,29,51^. *Arabidopsis* BBX32 (AtBBX32) interacted with soybean BBX62 (GmBBX62) through the N-terminal B-box region of BBX32^52^. Those data suggest the B-box domain plays an essential and conservative role in heterodimerization, but the detailed molecular mechanism for the functioning of BBX domain is still not fully revealed. In this study, the interaction between BBX28 and CO (BBX1) provided another evidence to support heterodimerization of BBX proteins (Fig. 3). In addition, we further showed that several conserved cysteines in the N-terminus of BBX28, particularly those within the B-box domain, are essential for BBX28-CO interaction and the activation of CO on *FT* transcription (Fig. 5). The structural basis for the role of heterodimerization between BBX28 and CO under light/dark condition in flowering regulation will be of great interest to be investigated in the future.

The evolutionary and structural relationships of BBX proteins have been investigated in many plant species, from algae to monocots and dicots, by the phylogenetic approach^53^. *Heading date 1*(*Hd1*), the ortholog of *CO* in rice, activates flowering under SD while delays flowering time under LD^35^. OsBBX27 (or OsCO3) and OsBBX5 (or OsCOL4) are negative regulators in plant flowering regulation^36,37^. Overexpressing Os*BBX14* or Os*COL9* under both SD and LD delayed the heading date in rice by inhibiting the expressions of florigen genes^38,39^. CmBBX8, a presumed chrysanthemum homolog of *Arabidopsis* BBX8 (*At*BBX8), accelerated flowering by directly targeting Cm*FLT1*, a floral inducer gene^40^. However, CmBBX13 acted as a flowering repressor independently of the photoperiodic pathway^41^. Here we found overexpressing *BBX28* delays flowering under LD through CO and *FT* (Figs. 1 and 6, S5). The delayed flowering of *BBX28*OE × *CO*OE plants is comparable to that of *CO*OE plants and the accordant dose curve of *CO* and *FT* transcript levels in *BBX28* and *CO* co-overexpressing plants (Figs. 6d, S3b) supported that BBX28 functions in flowering through CO and *FT*. Therefore, a new flowering regulator, BBX28, was identified in this study.

BBX28 was known to negatively regulate photomorphogenesis by interacting with ELONGATED HYPOCOTYL 5 (HY5) and interfering with the activity of HY5 in its downstream target gene expression. Besides, COP1 interacts with BBX28 and mediates its protein degradation in darkness via the 26S proteasome, demonstrating key roles of BBX28 in COP1-HY5 axis to maintain proper HY5 activity and normal photomorphogenic development^42^. We showed that BBX28 interacts with CO and decreases the recruitment of CO to *FT* promoter and weakens the activation of CO on *FT* transcription. The BBX28-HY5 axis in photomorphogenesis and BBX28-CO-FT axis in flowering regulation indicated that BBX28 plays roles in two different light signaling pathways through diverse interacting proteins to regulate downstream gene expression. It was shown that COP1 interacts with BBX28 and CO and mediates their degradations in darkness^9,42^. The co-localizations of these three proteins in photobodies suggested that BBX28, COP1 and CO may act in the same complex (Fig. S6a). Genetic analyses indicated that the negative role of BBX28 in flowering is CO-dependent under LD (Figs. 6, S5). CO was shown to act genetically downstream of COP1 to regulate flowering^9^. It is therefore of interest to study if and how BBX28 affects COP1-mediated CO degradation in flowering when these three proteins interact with each other. Additionally, we found *CO* transcription was not affected by BBX28 during the day but decreased about half in *BBX28*OE plants after dusk (Fig. 2c). It is also of interest to explore the relationship between *BBX28* and *CO* at the transcriptional level.

We proposed a working model for BBX28 in flowering regulation (Fig. S6b). CO binds to *FT* promoter to activate *FT* transcription. The interaction between N-terminus of BBX28 and CO decreases the recruitment of CO to *FT* locus. The N-terminal cysteines play an indispensable role in BBX28-CO heterodimerization and activation of CO on *FT* transcription. In wild type (WT/Col-0), the balance between BBX28 and CO maintains precise *FT* expression, which leads to normal flowering. When *BBX28* is over-expressed (*BBX28*OE), the recruitment of CO on *FT* locus is decreased by the overdosed BBX28 protein, resulting in a significant reduction of *FT* transcript which delays flowering under LD. Considering the similar protein structures of B-box transcription factors, the working model of BBX28 presented here might be applied to other BBX proteins in some degree. In addition, as BBX proteins have roles not only in flowering regulation, but also in photomorphogenesis, shade-avoidance response, abiotic and biotic stresses and plant hormonal pathways^27^, it is therefore of interest to study if and how the working model of BBX28 applies to those signaling pathways.

## Methods

### Plant materials and growth conditions

*Arabidopsis thaliana* ecotype Columbia-0 (Col-0) was used as the wild type for all experiments. The T-DNA insertion mutants of *bbx28* (SAIL_828_G11) and *co* (*co-9*, SAIL_24_H04) were obtained from the Arabidopsis Biological Resource Center (ABRC), and the homozygous lines were identified by PCR using the primers listed in Table S1. The seeds were surface sterilized and plated on Murashige and Skoog (MS) plates. After stratification for 4 days in dark at 4 °C, the plates were transferred to the growth chambers at 22 °C with different light conditions based on experimental requirements. The long day (LD) condition was 16 h light/8 h dark.

### Yeast two-hybrid assays

Yeast two-hybrid interaction assays were performed according to the Yeastmaker Yeast Transformation System 2 User Manual (Clontech). The full lengths of CDS, truncated fragments or mutated sequences of genes of interest were subcloned into pGADT7 or pGBKT7, respectively. The constructs were then co-transformed into yeast (AH109) according to the user manual. The yeast cells containing the bait and the prey constructs were grown on selective plates (SD-Leu-Trp, SD-Leu-Trp-His, SD-Leu-Trp-His-Ala and SD-Leu-Trp-His + 3-AT) for analysis. The concentration of 3-AT was 3 mM or 10 mM.

### Microscopy

Tobacco (*N. tabacum*) leaves were used for transient expression assays. The constructs were introduced into *Agrobacterium tumefaciens* strain GV3101 by electroporation and infiltrated into tobacco leaves with an injection syringe. 48 h later, the infiltrated tobacco leaf disks were subjected to microcopy analyses as described previously. Image stacks of nuclei were subjected to deconvolution by using softWoRx software (Applied Precision)^54–56^. Co-localization or Nucleolus-tethering System (NoTS) assays were performed as previously described^43^.

### Firefly luciferase (LUC) complementation imaging assays

The firefly luciferase (LUC) complementation imaging assay was performed as previously described^57^. BBX28, BBX28NT (1–112 amino acids, aa), BBX28CT (113–223 aa) and CO were fused with the N- or C-terminal fragment of LUC (NLuc and CLuc) respectively. The fused plasmids were introduced into GV3101 by electroporation and then co-infiltrated into tobacco (*N. benthamiana*) leaves with an injection syringe. 48 h later, the infiltrated leaves were injected with 100 mM luciferin (Sango, dissolved in water) and the luciferase signals were detected by the PMCapture software (Version 1.00) of a Chemiluminescence Imaging System (Tanon 5500, Shanghai, China).

### Co-immunoprecipitation (Co-IP) assays

The plasmid pairs for BBX28-CO interaction assays were co-infiltrated into tobacco (*N. benthamiana*) leaves. 48 h later, the leaves were collected and ground into good powder in liquid nitrogen for nuclear protein extraction. Samples were re-suspended in 20 ml CLB1 (50 mM HEPES, pH7.5, 150 mM NaCl, 1 mM EDTA, 0.04% (v/v) β-mercaptoethanol, 1% (v/v) Triton X-100, 10% (v/v) glycerol, 1 × Cocktail) and incubated at 4℃ for 30 min. The suspensions were filtrated through a double layer of Miracloth (Millipore) twice and centrifuged at 3000×*g* for 20 min at 4 ℃. The pellets were washed twice with 1 ml CLB2 (50 mM HEPES, pH7.5, 150 mM NaCl, 1 mM EDTA, 1% (v/v) Triton X-100, 10% (v/v) glycerol, 1 × Cocktail) and re-suspended in 200 µl CLB2 added with 20 µl 10% (w/v) SDS. The suspensions were sonicated five times and centrifuged at 12,000×*g* for 10 min at 4 °C. Another 1.8 ml CLB2 buffer was added for resuspension. The nuclear proteins were incubated with 50 µl Anti-GFP mAb-Magnetic Beads (MBL) at 4℃ for 4 h with rotation. The beads were washed 5 times with washing buffer (50 mM HEPES, pH7.5, 150 mM NaCl, 10% (v/v) glycerol, 0.1% (v/v) TritonX-100, 1 mM EDTA, 1 × Cocktail). The proteins were released by boiling at 100 °C for 5 min and subjected to Western blotting assays with anti-GFP (Abmart; M20004) and anti-mCherry (Abcam; ab67453) antibodies. Blotting signals were detected by the PMCapture software (Version 1.00) of a Chemiluminescence Imaging System (Tanon 5500, Shanghai, China).

### Transgenic plants

The constructs were introduced into GV3101 by electroporation and transformed into Col-0 by the floral-dip method^58^. To obtain transgenic plants in different mutant backgrounds, the plants in Col-0 background were crossed with mutants and the backgrounds were identified by PCRs. Primers used were shown in Table S1.

### Site-direct mutagenesis

Site-direct mutagenesis was generated by the two-step overlap PCR. We designed a pair of mutant primers by artificially changing the base sequences of interest. When introducing cysteine to alanine (Cys-to-Ala) substitutions, we changed the bases to GCC. Then PCRs were performed by using the wild type forward primer and mutated reverse primer or the mutated forward primer and wild type reverse primer. The same amounts of the purified PCR products were mixed and used as the template for final PCR, which was performed by using the wild type forward and reverse primers. The site-direct mutagenesis was confirmed by sequencing. Primers used were shown in Table S1.

### Flowering time and rosette leaf counting

After surface sterilization and stratification for 4 days in dark at 4 °C, the seeds on plates were transferred to the growth chambers at 22 °C under LD (16 h light/8 h dark) for 7 days. Then the seedlings were transferred from the plates to the soil and grown in the greenhouse at 22 °C under LD. The flowering times and rosette leaf numbers were recorded when plants start bolting.

### Quantitative real-time PCR

Expression analyses were performed by quantitative real-time PCR (qRT-PCR) as described previously^43^. Total RNAs were extracted from 7-day-old seedlings under the LD condition using the RNeasy Plant Mini Kit (Qiagen). RNAs (about 1 µg) were used as templates for reverse transcription using ReverTra Ace qPCR RT Master Mix with gDNA Remove Kit (Toyobo) according to the manufacturer’s instruction. The cDNAs were then diluted and used as templates for qPCR using SYBR Premix Ex Taq (TliRNaseH Plus) in a Bio-Rad CFX96 real-time system. All PCRs were performed by preincubation for 2 min at 95 °C, followed by 50 cycles of denaturation at 95 °C for 20 s, annealing at 56 °C for 30 s, and extension at 72 °C for 30 s. Each reaction was repeated three times. Data were captured and analyzed by Bio-Rad CFX Manager software (Version 2.1) (Bio-Rad). *Actin2* (*At3g18780*) was used for data normalization. Primers used for qRT-PCR were listed in Table S1.

### Protein extraction and immunoblot assays

For total proteins of plants or seedlings grown under different conditions were extracted by extraction buffer (50 mM Tris–HCl, pH7.5, 150 mM NaCl, 10 mM MgCl2, 5 mM EDTA, 10% (v/v) Glycerol, 0.6 mM PMSF, 1 × Cocktail, Roche) (200 µl extraction buffer for about 50 µl powder). Lysed proteins were placed on ice for 20 min and mixed several times. Then proteins were centrifuged at 14,000×*g* for 10 min at 4 °C. The supernatants were transformed to new tubes and 5 × SDS-PAGE loading buffer (250mMTris-HCl, pH 6.8, 10% (w/v) SDS, 0.5% (w/v) BPB, 50% (v/v) Glycerol, 5% (v/v) β-mercaptoethanol) was added. Samples were boiled at 100 °C for 5 min and subjected to SDS-PAGE for Western blotting assays. Blotting signals were detected by the PMCapture software (Version 1.00) of a Chemiluminescence Imaging System (Tanon 5500, Shanghai, China).

### Dual-luciferase (dual-LUC) assay

The Dual-LUC assay was performed as described previously^59^. The *FT* promoter (1800 bp) was subcloned into pGreenII 0800-LUC vector (a kind gift from Prof. Hongtao Liu) as the reporter and full-length of *BBX28* CDS, *BBX28* truncates or point mutations and full-length of *CO* CDS were subcloned into pCambia131-35S-N1-YFP^54^ as the effectors. pCambia131-35S-YFP (YFP) was used as the control effector. The reporter construct was introduced into the GV3101 harboring pSoup-P19. The effecter constructs were introduced into GV3101. The cells were harvested when grown to OD600 = 0.8–1.0 and washed once and then resuspended by sterilized water to OD600 = 0.8. The reporter and the effecter were mixed together at 1:2 ratio for co-infiltration of the tobacco leaves. For assays containing two effectors, the reporter and effectors were mixed together at 1:2:2 ratio. The control and the experimental samples were infiltrated into the same tobacco leaf and more than three independent leaves were served as technical replicates. 48 h later, the tobacco leaf disks in the infiltrated areas were collected by a puncher with 1.1 cm in diameter for protein extraction. Extracted proteins were subject to Dual-LUC assays by the Dual-Luciferase Reporter Assay System (Promega E1910) with GloMax 20/20 Luminometer software (version 1.10) (Promega).

The samples were ground in liquid nitrogen in 1.5 ml EP tubes and extracted by 100 µl 1 × Passive Lysis Buffer (5 ×), incubated on ice for 15 min and mixed several times for efficient lysis. The extracted proteins were centrifuged at 14,000×*g* for 10 min at 4 °C and supernatants were transformed into new tubes. 8 µl supernatant was transformed to a new tube and 40 µl luciferase assay buffer was added and mixed for recording the LUC value. Then 40 µl Stop&Glo buffer was added and mixed for recording the REN value. The LUC/REN ratios were calculated. Data from the luminometer were exported by the GloMax Spreadsheet Interfacer (Promega).

### Chromatin immunoprecipitation (ChIP) assay

ChIP assay was performed as previously described^60^. 1 g 12-day-old seedlings of *CO*OE-2# (*35S-CO-YFP*/Col-0) and *BBX28*OE × *CO*OE-3# (*35S-BBX28-3* × *FLAG-mCherry*/Col-0 × *35S-CO-YFP*/Col-0) were harvested at ZT12 under LD and cross-linked twice in fixing buffer (0.4 M Sucrose, 10 mM Tris–HCl, pH8.0, 1 mM EDTA, 1% Formaldehyde) for 10 min by vacuum infiltration. The cross-linking was terminated by the addition of 2.5 M Glycine and vacuum infiltration for 5 min. The samples were ground into good powder, then the nuclei and chromatin were isolated as described in IP assays. 5 µg anti-GFP antibody (Abcam) was incubated with 20 µl Protein A + G magnetic ChIP beads (Millipore) at 4 °C for 3–5 h following by the addition of extracted chromatins and incubated at 4 °C overnight. Then the beads were washed by low salt washing buffer (50 mM HEPES, pH7.5, 1 mM EDTA, 150 mM NaCl), high salt washing buffer (50 mM HEPES, pH7.5, 1 mM EDTA, 500 mM NaCl), LiCl wash buffer (10 mM Tris–HCl, pH8.0, 1 mM EDTA, 0.25 M LiCl, 0.5%(v/v) NP-40) and TE buffer (10 mM Tris–HCl, pH8.0, 1 mM EDTA) and eluted with elution buffer (1% SDS, 0.1 M NaHCO_3_). After overnight reverse cross-linking, DNA was purified and dissolved in 30 µl water. Four fragments (FT1, FT2, FT3 and FT4) of *FT* locus were used for ChIP qRT-PCR assay^17^. Data were captured and analyzed by Bio-Rad CFX Manager software (Version 2.1) (Bio-Rad). The ChIP results were reported by fold enrichment way using 2^−△△Ct^ method^61^. GFP-IP signal relative to corresponding nonimmune control (NIC; no GFP antibody) signal was normalized by FT4. Primers used were listed in Table S1.

### Transcriptional activity assay

The transcriptional activity assay was performed as previously described with some modifications^62^. The original plasmids were a kind gift from prof. Zhukuan Cheng’s lab. The construct 35S’-5 × Gal4-TATA-LUC-Nos was used as the reporter and CaMV35S-Ω-Gal4BD-Nos as the effecter. Gal4BD-VP16 was the positive control and pTRL was the internal reference. The 35S’-5 × Gal4-TATA fragment was amplified by the KpnI-35S’-F and KpnI-TATA-R primers using 35S’-5 × Gal4-TATA-LUC-Nos as the template and subcloned into pGreenII 0800-LUC vector to generate pGreenII 0800-35S’-5 × Gal4-TATA-LUC plasmid. The Gal4BD sequence was amplified by BamHI-Gal4BD-F and BamHI-Gal4BD-R primers using CaMV35S-Ω-Gal4BD-Nos as the template and subcloned into pCambia131-35S-N1-YFP to generate pCambia131-35S-Gal4BD-N1-YFP. To construct different effectors, the coding sequences of different genes were subcloned into pCambia131-35S-Gal4BD-N1-YFP plasmid. For the positive control (pCambia131-35S-Gal4BD-VP16-YFP), the VP16 sequence was amplified by SpeI-VP16-F and SpeI-VP16-R primers using Gal4BD-VP16 as the template and subcloned into pCambia131-35S-Gal4BD-N1-YFP. Then constructs of the reporter and effectors were introduced into GV3101 harboring pSoup-P19 or GV3101 and co-infiltrated into tobacco (*N. benthamiana*) leaves for Dual-LUC assay by the Dual-Luciferase Reporter Assay System (Promega E1910) with GloMax 20/20 Luminometer software (version 1.10) (Promega) to analyze the transcriptional activities. Data from the luminometer were exported by the GloMax Spreadsheet Interface software (Promega).

### Histochemical GUS staining

The promoter of *BBX28* (2004 bp) was subcloned into pBI101.1-GUS vector and transformed into Col-0 to generate p*BBX28-GUS*/Col-0 transgenic plants. 10-day-old LD grown seedlings were soaked in GUS staining buffer (1 mg/ml 5-bromo-4-chloro-3-indolyl-beta-D-glucuronic acid cyclohexylammonium salt (X-Gluc), 50 mM Na_3_PO_4_ (pH7.0), 0.1% (v/v) Triton X-100, 2 mM K_4_Fe (CN)_6_·3H_2_O, 2 mM K_3_Fe (CN)_6_ and 10 mM EDTA) at 37 °C overnight in darkness. 75% ethanol was used to remove chlorophyll for imaging.

### Statistical analyses

For quantitative real-time PCR, flowering time and rosette leaf counting, Dual-LUC assay and transcriptional activity assay, the differences between samples were analyzed by student’s t-test. For all analyses, statistical significance was set as ****p < 0.0001, ***p < 0.001, **p < 0.01, *p < 0.05, and ns (not significant).

## Supplementary information

Supplementary Information.

## Data Availability

All necessary data generated or analyzed during the present study are included in this published article and its Supplementary Information files.
