# Supplementary material for: B-box transcription factor 28 regulates flowering by interacting with constans
Source: Sci Rep. 2020 Oct 20;10:17789. doi: 10.1038/s41598-020-74445-7 (PMC7575571; doi:10.1038/s41598-020-74445-7)
Supplement: Supplementary file 1 — Supplementary Information. [file 41598_2020_74445_MOESM1_ESM.pdf]

B-BOX TRANSCRIPTION FACTOR 28 regulates flowering by interacting with CONSTANS

Yin Liu[1,2], Guang Lin[2], Chunmei Yin[1], and Yuda Fang[1,2]***

[1]Joint Center for Single Cell Biology, School of Agriculture and Biology, Shanghai Jiao Tong University, Shanghai 200240, China

[2]National key Laboratory of Plant Molecular Genetics, CAS Center for Excellence in Molecular Plant Sciences, Institute of Plant Physiology and Ecology, Chinese Academy of Sciences; University of Chinese Academy of Sciences, Shanghai 200032, China

***Address correspondence to yuda.fang@sjtu.edu.cn**

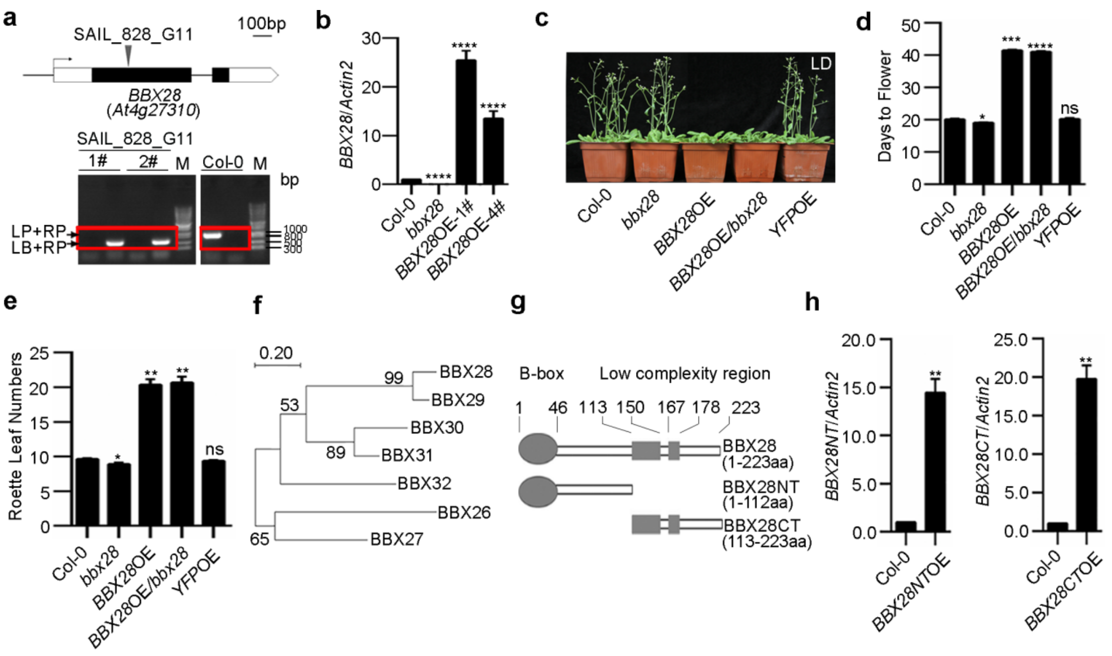

Figure S1. Characterization of *bbx28* mutant, and overexpressing lines of *BBX28*, *BBX28NT* and *BBX28CT*.

(**a**) Scheme and identification of *bbx28* T-DNA insertion mutant (SAIL_828_G11). White boxes indicate the 5’ untranslated region (5’ UTR) and 3’UTR. Black boxes indicate exons. The region (dark line) between two exons is the intron. Dark triangle indicates the T-DNA insertion position. LB indicates the primer located in the T-DNA sequence (Salk Institute Genomic Analysis Laboratory (SIGnAL); http://signal.salk.edu/tdnaprimers.2.html). LP and RP indicate the left and the right primers located in *BBX28* genomic DNA respectively. 1# and 2# are two SAIL_828_G11 plants. LP+RP primers produce a band with 974bp in Col-0 but not in 1# and 2#, however LB+RP primers produce a band between 439 and 739bp (predicted by SIGnAL) in 1# and 2# but not in Col-0, suggesting 1# and 2# are homozygous T-DNA insertion mutants.(**b**) Transcript levels of *BBX28* in Col-0, *bbx28* or *BBX28* overexpressing plants (*BBX28*OE: *35S-BBX28-YFP*/Col-0; n=4 biological replicates). Numerals with the pound sign represent independent lines. (**c**) Visual phenotypes of 33-day-old Col-0, *bbx28*, *BBX28*OE, *BBX28*OE/*bbx28* and *YFP*OE plants under LD. *YFP*OE: *35S-YFP*/Col-0. (**d**) and (**e**) Flowering time and rosette leaf numbers of genotypes in (**c**) (n=3 biological replicates; plant number=10 in each replicate). (**f**) Phylogenetic tree of group V BBX proteins (BBX26-BBX32) by

Molecular Evolutionary Genetics Analysis (MEGA). (**g**) Schemes of the domain structure of BBX28 and its truncates. Ellipse indicates the B-box domain. Filled rectangles indicate low-complexity regions and empty rectangles indicate proteins sequence without special domains. Numbers with gray lines indicate the amino acid positions. (**h**) *BBX28NT* or *BBX28CT* transcript levels in Col-0, *BBX28NT* or *BBX28CT* overexpressing plants (n=4 for biological replicates). *BBX28NT*OE: *35S-BBX28NT-YFP*/Col-0; *BBX28CT*OE: *35S-BBX28CT-YFP*/Col-0). In (**b**) and (**h**), plants were grown under LD for 7 days and subjected to quantitative real-time PCR (qRT-PCR) assay. *Actin2* was used for data normalization. Data are means ± SEM. Statistical significance was analyzed by student's *t*-test; ****p<0.0001, ***p<0.001, **p<0.01, *p<0.05; ns, not significant.

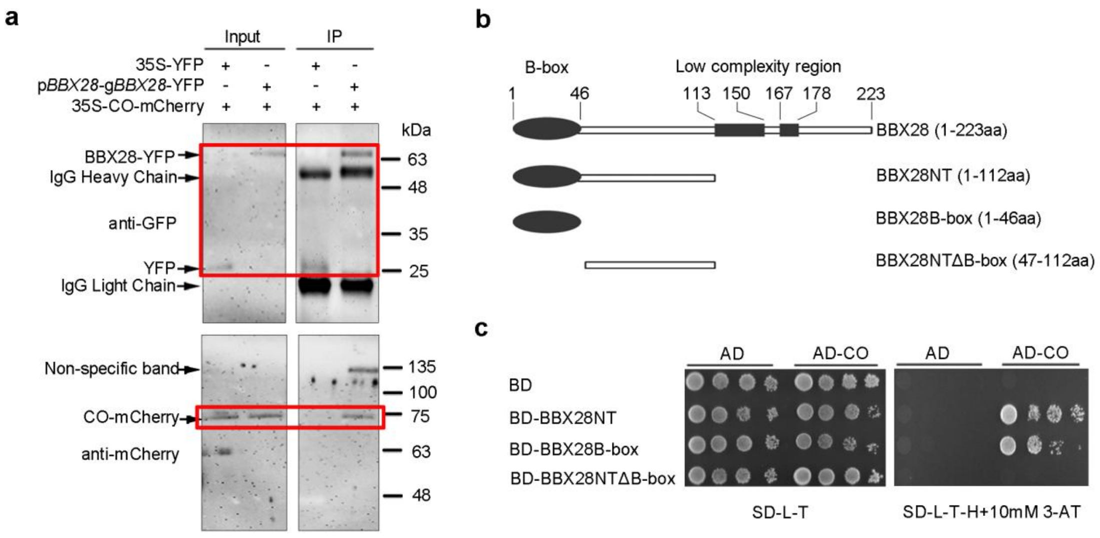

Figure S2. The B-box domain within BBX28 N-terminus interacts with CO.

(a) The full-length blots of Co-IP assay of BBX28-CO interaction. Tobacco leaves co-expressing CO-mCherry and YFP acted as the negative control. Proteins were detected by western blots with anti-GFP and anti-mCherry antibodies. Molecular weight standards are indicated. The cropped micrographs are shown in Figure 3e. (**b**) Schemes of BBX28 B-box and BBX28NTΔB-box truncations. Ellipse indicates the B-box domain. Filled rectangles indicate low-complexity regions and empty rectangles indicate proteins sequence without special domains. Numbers with gray lines indicate the amino acid positions. (**c**) Yeast two-hybrids between CO and BBX28B-box or BBX28NTΔB-box. Cells were grown on selective plates for interaction assays.

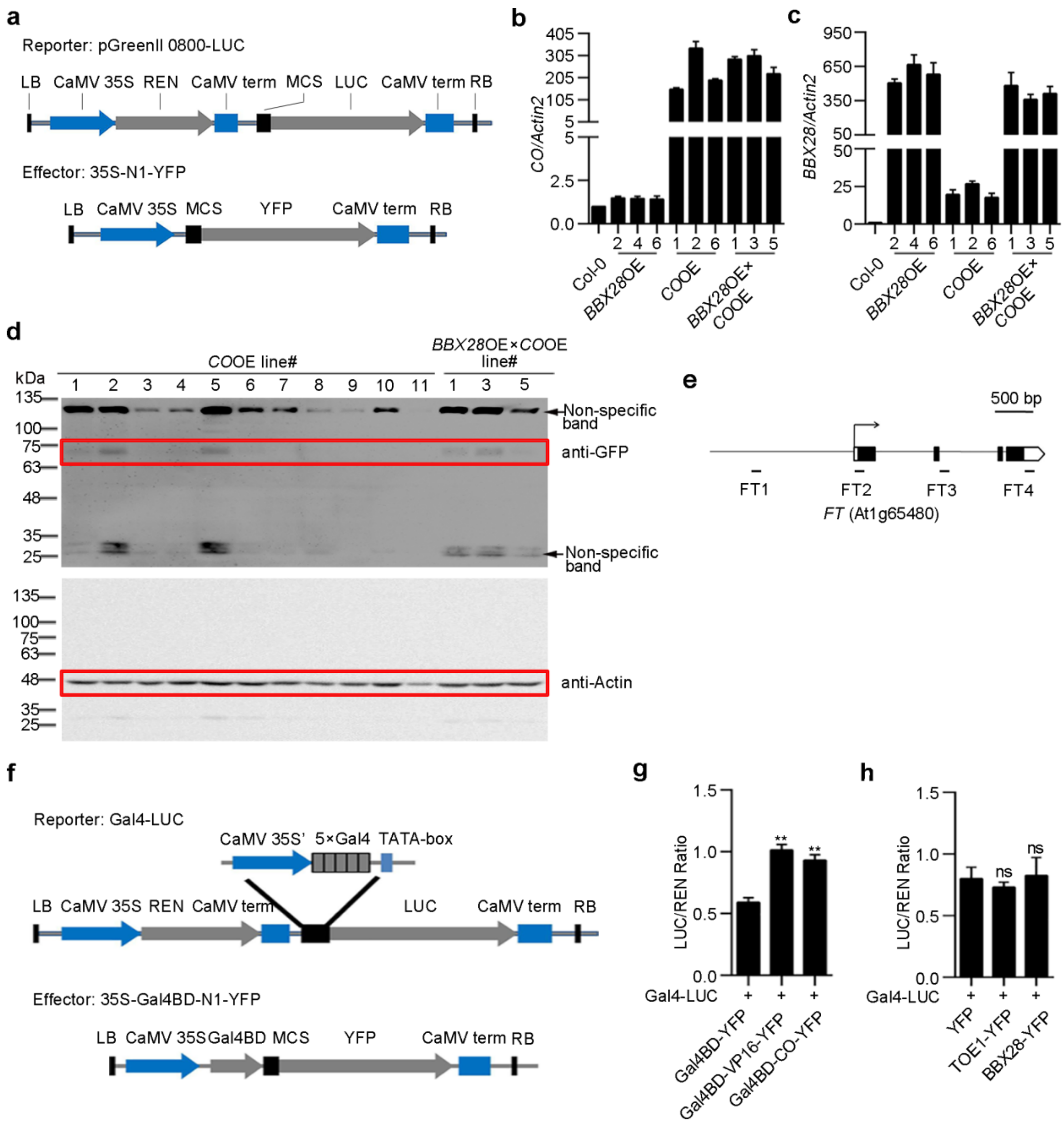

Figure S3. BBX28 inhibits CO targeting to *FT* locus without affecting the transcriptional activation activity of CO.

(**a**) Scheme of Dual-LUC assay system. 35S promoter (CaMV 35S), 35S terminator (CaMV term), Renilla luciferase (REN), firefly luciferase (LUC), YFP coding sequence (YFP), multiple cloning sites (MCS) and T-DNA left border (LB) and right border (RB) are indicated. (**b**) and (**c**) qRT-PCR analyses of *CO* (**b**) and *BBX28* (**c**) expressions in 7-day-old LD grown Col-0, *BBX28*, *CO* overexpressing plants or *BBX28* and *CO* co-overexpressing plants. *CO*OE: *35S-CO-YFP*/Col-0; *BBX28*OE: *35S-BBX28-3×FLAG-mCherry*/Col-0. Numerals represent independent lines. Data are mean± SD (n=3 technical replicates of each plant). (**d**) The full-length blots of CO protein levels in *CO*OE and *BBX28*OE×*CO*OE plants. 7-day-old LD grown seedlings

were harvested at ZT12 and subjected to immunoblot assay. Proteins were detected by western blotting with anti-GFP and anti-Actin antibodies. The bands in red boxes represent specific signals detected. Actin served as the loading control. Numbers indicate different lines. Molecular weight standards are indicated. (**e**) Scheme showing the positions of ChIP-qPCR amplicons in *FT* (FT1, FT2, FT3 and FT4). Filled and empty rectangles indicate exons and untranslated regions respectively. (**f**) Schemes of the transcriptional activity assay system. CaMV35S promoter (CaMV 35S), 35S terminator (CaMV term), Renilla luciferase (REN), firefly luciferase (LUC), 35S promoter without TATA-box (CaMV 35S'), five repeats of Gal4 sequence (5×Gal4), TATA-box and T-DNA left border (LB) and right border (RB) are indicated in the reporter vector Gal4-LUC. Gal4 DNA binding domain (Gal4BD), multiple cloning sites (MCS) and YFP coding sequence (YFP) are indicated in the effector vector (35S-Gal4BD-N1-YFP). (**g**) Transcriptional activity assay of CO. Gal4BD-YFP served as a negative control. Gal4BD-VP16-YFP served as a positive control. (**h**) The transcriptional activity assays show the effects of YFP, BBX28 and TOE1 on Gal4-LUC expression. YFP served as a negative control. In (**g**) and (**h**), n=3 biological replicates. Data are means ± SEM. Statistical significance was analyzed by student's *t*-test; ***p<0.01; ns, not significant.

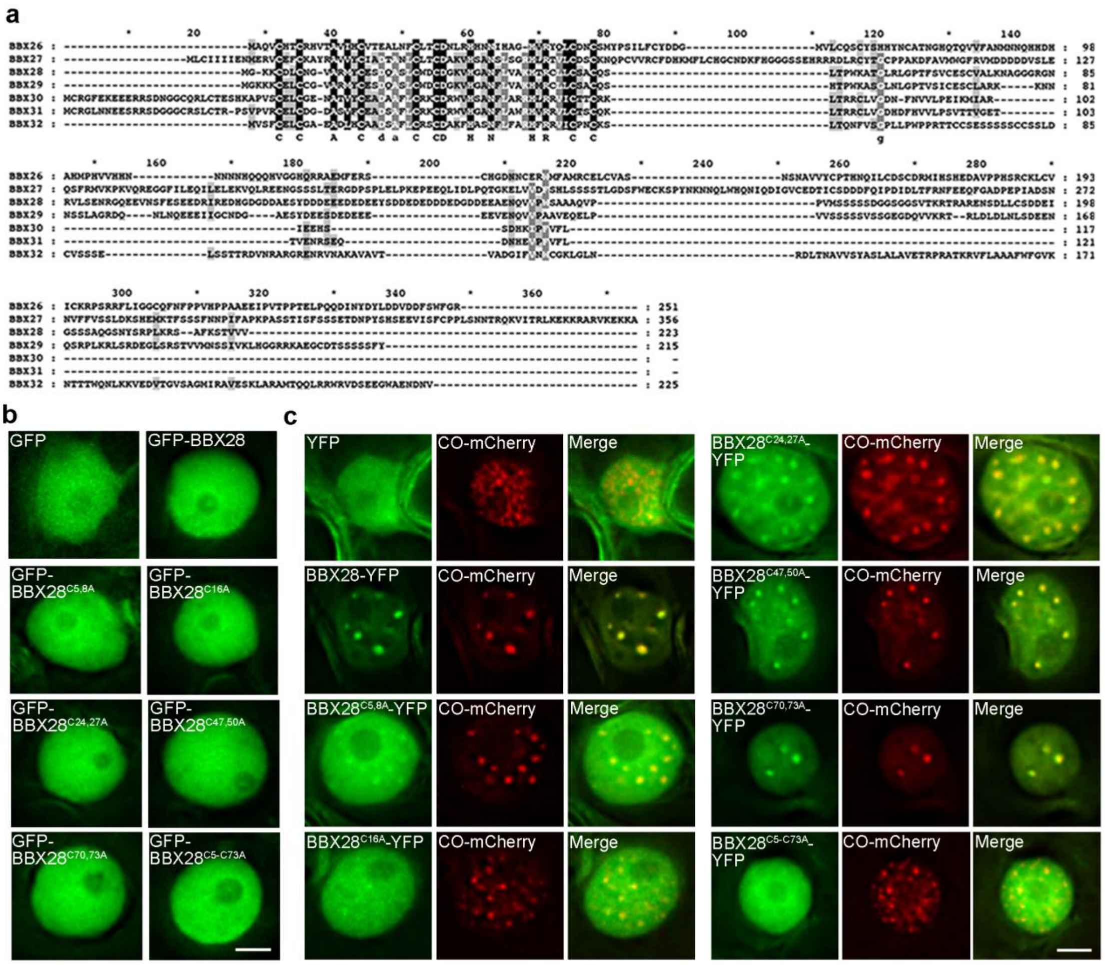

Figure S4. The effects of BBX28 N-terminal cysteines on BBX28 subcellular distribution and colocalization with CO.

(**a**) Conserved cysteines located in the N-terminus of Group V B-box family members. The protein sequences of BBX26-BBX32 were subjected to multiple alignments by Clustal Omega of the European Bioinformatics Institute (EMBL-EBI) (http://www.ebi.ac.uk/Tools/msa/clustalo/). (**b**) Subnuclear distributions of BBX28 cysteine mutants in tobacco leaves. GFP served as a control. Bar =5μm. (**c**) Co-localization analysis of CO and BBX28 cysteine mutants in tobacco leaves. YFP served as a control. Bar =5μm.

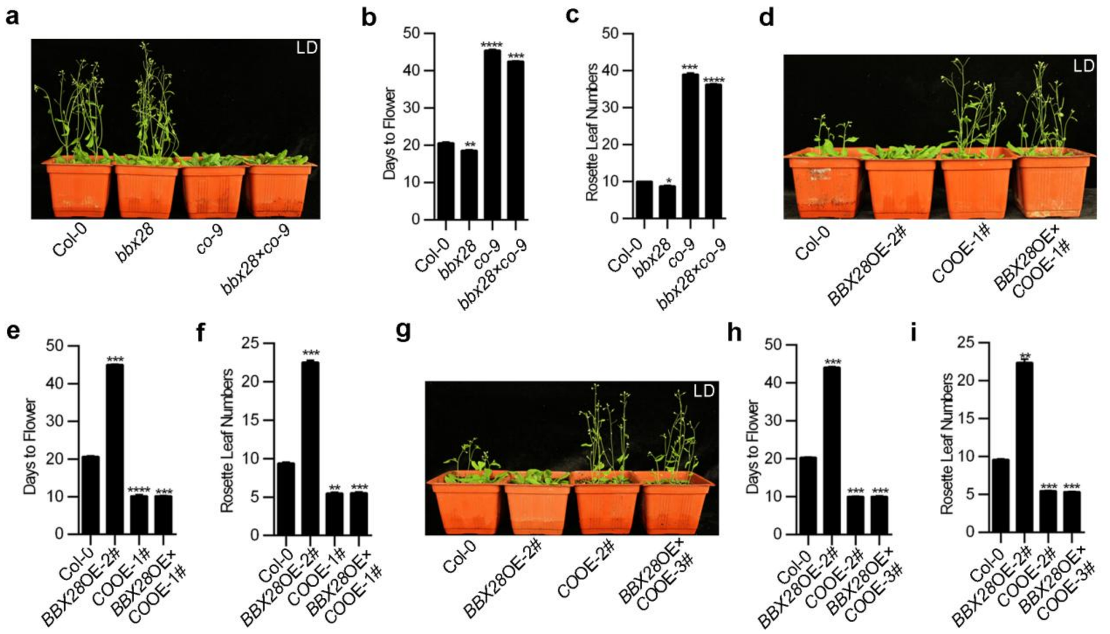

Figure S5. BBX28 regulates flowering through CO and *FT*.

(**a**) Visual phenotypes of 36-day-old Col-0, *bbx28*, *co-9* and *bbx28×co-9* plants under LD. (**b**) and (**c**) Flowering time and rosette leaf numbers of different genotypes in (**a**). Data are means ± SEM (n=3 biological replicates; plant numbers=8 in each replicate). (**d**) Visual phenotypes of 27-day-old Col-0, *BBX28* overexpressing (2#), *CO* overexpressing (1#) or *BBX28* and *CO* co-overexpressing (1#) plants under LD. *CO*OE: *35S-CO-YFP*/Col-0; *BBX28*OE: *35S-BBX28-3×FLAG-mCherry*/Col-0. Numerals with the pound sign represent independent lines. (**e**) and (**f**) Flowering time and rosette leaf numbers of different genotypes in (**d**). (**g**) Visual phenotypes of 27-day-old Col-0, *BBX28* overexpressing (2#), *CO* overexpressing (2#) or *BBX28* and *CO* co-overexpressing (3#) plants under LD. (**h**) and (**i**), Flowering time and rosette leaf numbers of different genotypes in (**g**). In (**e**), (**f**), (**h**) and (**i**), data are means ± SEM (n=3 biological replicates; plant numbers=12 in each replicate). Statistical significance was analyzed by student's *t*-test; ****p<0.0001, ***p<0.001, **p<0.01, *p<0.05.

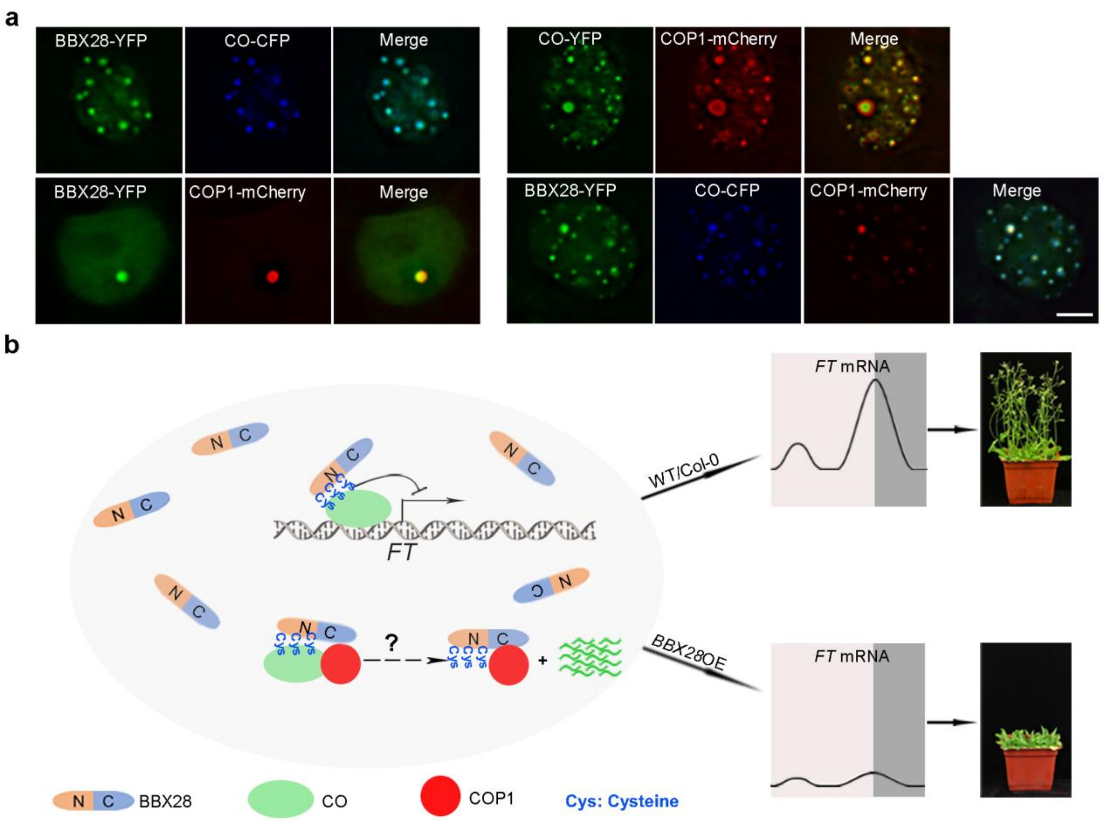

Figure S6. A model for the negative role of BBX28 in flowering regulation.

(**a**) Co-localizations among BBX28, COP1 and CO in tobacco leaves. Bar =5μm. (**b**) A model for the role of BBX28 in repressing flowering under LD. CO binds to *FT* promoter to activate *FT* transcription. The interaction between N-terminus of BBX28 and CO decreases the recruitment of CO to *FT* locus. The N-terminal cysteines play an indispensable role in BBX28-CO heterodimerization and activation of CO on *FT* transcription. In wild type (WT/Col-0), the balance between BBX28 and CO maintains precise *FT* expression, which leads to normal flowering. When *BBX28* is over-expressed (*BBX28*OE), the recruitment of CO to *FT* locus is decreased by an overdosed BBX28 protein, resulting in a significant reduction of *FT* transcript which delays flowering under LD. In addition, as co-localizations among BBX28, COP1 and CO were observed, if and how COP1 plays a role in this process is still unknown.

Table S1. Primers used in this study.

Primer Name	Primer Sequence(5'to 3')	Vector
**For yeast two-hybrid**		
BamHI-AT-AD/BD-BBX28-F	NNNGGATCCATATGGGGAAGAAGTGTGATTTATGTA	pGADT7 or pGBKT7
BamHI-AD/BD-BBX28-R	NNNGGATCCTTAAACAACAACCGTTGATTTAAAC	
BamHI-AT-AD/BD-BBX28NT-F	NNNGGATCCATATGGGGAAGAAGTGTGATTTATGTA	
BamHI-AD/BD-BBX28NT-R	NNNGGATCCTTAACCGTGATCTTCTCTAATCCGA	
BamHI-AT-AD/BD-BBX28CT-F	NNNGGATCCATATGGACGGTGACGACGCGGAGTCTT	
BamHI-AD/BD-BBX28CT-R	NNNGGATCCTTAAACAACAACCGTTGATTTAAAC	
EcoRI-AD/BD-BBX28B-box-F	NNNGAATTCATGGGGAAGAAGTGTGATTTATGT	
EcoRI-AD/BD-BBX28B-box-R	NNNGAATTCTTAGAGAAGACAACGCGTGTGTTT	
EcoRI-AD/BD-BBX28NTΔB-box-F	NNNGAATTCATGTGTAGCGCTTGTCAGTCTCTT	
EcoRI-AD/BD-BBX28NTΔB-box-R	NNNGAATTCTTAACCGTGATCTTCTCTAATCCG	
EcoRI-AD-CO-F	NNNGAATTCATGTTGAAACAAGAGAGTAACGAC	
EcoRI-AD-CO-R	NNNGAATTCTCAGAATGAAGGAACAATCCCATA	
**For subcellular localization, NoTS, Co-IP assays or transgenic plant construction**		
SalI-CO-YFP/CFP/mCherry-F	NNNGTCGACATGTTGAAACAAGAGAGTAACGAC	pCambia131-35S-N1-YFP/CFP/mCherry
SpeI-CO-YFP/CFP/mCherry-R	NNNACTAGTGAATGAAGGAACAATCCCATATCCT	
SalI-COP1-mCherry-F	CCCGTCGACATGGAAGAGATTTCGACGGATCCGG	pCambia131-35S-N1-mCherry
SpeI-COP1-mCherry-R	CCCACTAGTCGCAGCGAGTACCAGAACTTTG	
SalI-BBX28-YFP/CFP-F	NNNGTCGACATGGGGAAGAAGTGTGATTTATGTA	pCambia131-35S-Nuc2-linker-YFP; pCambia131-35S-N1-YFP/CFP
SpeI-BBX28-YFP/CFP-R	NNNACTAGTAACAACAACCGTTGATTTAAACG	
SalI-BBX28NT-YFP/CFP-F	NNNGTCGACATGGGGAAGAAGTGTGATTTATGTA	

SpeI-BBX28NT-YFP/CFP-R	NNNACTAGTACCGTGATCTTCTCTAATCCGA	
SalI-BBX28CT-YFP/CFP-F	NNNGTCGACATGGACGGTGACGACGCGGAGTCTT	
SpeI-BBX28CT-YFP/CFP-R	NNNACTAGTAACAACAACCGTTGATTTAAACG	
SacI-GFP-F	NNNGAGCTCATGGTGAGCAAGGGCGAGGAGCTGT	pCambia1300-35S-3×FLAG
SacI-GFP-R	NNNGAGCTCCTTGTACAGCTCGTCCATGCCGAG	
KpnI-BBX28-F	NNNGGTACCATGGGGAAGAAGTGTGATTTATGTA	
KpnI-BBX28-R	NNNGGTACCAACAACAACCGTTGATTTAAACG	
SalI-BBX28-F	NNNGTCGACATGGGGAAGAAGTGTGATTTATGTA	pCambia131-35S-N1-YFP; pBI101.1
SpeI-BBX28-3×FLAG-R	NNNACTAGTCTTATCGTCATCGTCCTTGTAATCGATGTCGTGATCCTTATAGTCTCCATCATGGTCTTTGTAGTC	
**For firefly luciferase complementation imaging assay**		
KpnI-BBX28-R	NNNGGTACCTTAAACAACAACCGTTGATTTAAA	CLuc
KpnI-BBX28NT-R	NNNGGTACCTTAACCGTGATCTTCTCTAATCCG	
KpnI-BBX28CT-F	NNNGGTACCATGGACGGTGACGACGCGGAGTCTT	
KpnI-CO-F	NNNGGTACCATGTTGAAACAAGAGAGTAACGAC	NLuc
SalI-CO-R	NNNGTCGACGAATGAAGGAACAATCCCATATCC	
**For ChIP–qPCR assay**		
FT1-F	GTGCAAATGGATGGTTAGTATTTTTAC	
FT1-R	CAAGCGGCCATATTATGGAAAAGTGAGTT	
FT2-F	CAGAAACAATCAACACAGAGAAACCA	
FT2-R	CTTGGCTTGTTTTGAACCTGAGAAGGCCT	
FT3-F	CAAGCCAGCCTTTAAGATACTC	

FT3-R	GGAAAATGAGATAACACAAG AAAG	
FT4-F	TGGTGCCATAGCTTAAACATG TG	
FT4-R	CATGTGAATTTTCTAGCTAGT AACT	
**For mutant identification**		
SAIL_828_G11-LP	CTTGTTTTGGAGATCGCAAAG	T-DNA mutant
SAIL_828_G11-RP	TATCCCAAATCCTGCAACTTG	
SAIL_24_H04-LP	AAGCTGTTGTGACACATGCTG	
SAIL_24_H04-RP	CCCCTTCTTTCAGATACCAGC	
LB1	GCCTTTTCAGAAATGGATAAA TAGCCTTGCTTCC	
**For Dual-LUC assay**		
HindIII-*pFT*-F	NNNAAGCTTACACTAACATGA TTGAATGACAAA	pGreenII 0800-LUC
HindIII-*pFT*-R	NNNAAGCTTCTTTGATCTTGA ACAAACAGGTGG	
KpnI-35S-F	NNNGGTACCAAGCTTGCATGC CTGCAGGTCCCC	pGreenII 0800-LUC
KpnI-TATA-R	NNNGGTACCGGTCGACTGTAA TTGTAAATAGTAAT	
BamHI-Gal4BD-F	NNNGGATCCATGAAGCTACTG TCTTCTATCGAA	pCambia131-35S-N1-YFP
BamHI-Gal4BD-R	NNNGGATCCCGATACAGTCA ACTGTCTTTGACC	
SpeI-VP16-F	NNNACTAGTACCGATGTCAGC CTGGGGGACGAG	pCambia131-35S-Gal4BD-N1-YFP
SpeI-VP16-R	NNNACTAGTCCCACCGTACTC GTCAATTCCAAG	
SalI-CO-F	NNNGTCGACATGTTGAAACA AGAGAGTAACGAC	
SpeI-CO-R	NNNACTAGTGAATGAAGGAA CAATCCCATATCCT	
SalI-BBX28-F	NNNGTCGACATGGGGAAGAA GTGTGATTTATGTA	
SpeI-BBX28-R	NNNACTAGTAACAACAACCG TTGATTTAAACG	
EcoRI-TOE1-F	NNNGAATTCATGTTGGATCTTA ACCTCAACGCT	pCambia131-35S-N1-YFP
SpeI-TOE1-R	NNNACTAGTAGGGTGTGGATA AAAGTAACCACG	
**For GUS staining**		

HindIII-*pBBX28*-F	NNNAAGCTTTGAATCAAAGACTTTGACATAA	pBI121
BamHI-*pBBX28*-R	NNNGGATCCTTTAATCAAAATCAAACTCTTT	
**For real-time PCR**		
qBBX28-F-1	GAGTCATGCGTCGCTCTTAAA	*bbx28* T-DNA
qBBX28-R-1	CGATCTTCTTCGGACTCGAAAC	
qBBX28-F-2	GAGGATGGTGATGATGAGGAAG	
qBBX28-R-2	CCTCCGTCAGAAGATGAAGAAC	
qActin2-F	GACCTTTAACTCTCCCGCTATG	
qActin2-R	GAGACACACCATCACCAGAAT	
qCO-F	CAACAGCTTCACACCCAAGAACG	
qCO-R	TTGCAGGGTCAGGTTGTTGCTC	
qFT-F	GCTACAACTGGAACAACCTTTGGC	
qFT-R	TGAATTCCTGCAGTGGGACTTGG	
qSOC1-F	TTCGCCAGCTCCAATATGCAAG	
qSOC1-R	TGCTGACTCGATCCTTAGTATGCC	
qLFY-F	TGATGCTCTCTCCCAAGAAGGG	
qLFY-R	TCAGTCTGGTCTTGTTGCTGCAC	
qFUL-F	TCGAATATTCCACCGACTCTTGC	
qFUL-R	TTTGTGAAACGTCTCGGCCAAC	
qSVP-F	AACGCTGCTGTGTACGAGGAAG	
qSVP-R	TCTCTAACCACCATACGGTAAGCC	
qTEM1-F	GGATGAATCCGTCTCCGACGAAAG	
qTEM1-R	AACCGCCACACTTTCCCTGTT	

	C	
qFLC-F	TGTTCAACTGGAGGAACACCTTG	
qFLC-R	AGCTTCAACATGAGTTCGGTCTTC	
qBBX28NT-F	TTGGGATTGCGACGGTAAA	
qBBX28NT-R	CGGCGTAAGAGACTGACAAG	
qBBX28CT-F	AAGAGGACGAGGGCTAGAG	
qBBX28CT-R	CTTCAACGGCCGAGAATAGT	
**For site-direct mutagenesis**		
BamHI-AT-AD/BD-BBX28^{C5,8A}-F	NNNGGATCCATATGGGGAAGAAGGCCGATTTAGCCAACGGTGT	pGADT7 or pGBKT7
KpnI-BBX28^{C5,8A}-F	NNNGGTACCATGGGGAAGAAGGCCGATTTAGCCAACGG	pCambia1300-35S-3×FLAG
KpnI-BBX28^{C16A}-F	NNNGGTACCATGGGGAAGAAGTGTGATTTATGTAACGGTGTTGCAAGAATGTATGCCGAGTCAGATCAAGCTAG	pCambia1300-35S-3×FLAG
BBX28^{C24,27A}-F	TAGTTTAGCCTGGGATGCCGACGGT	Overlap PCR
BBX28^{C24,27A}-R	ACCGTCGGCATCCCAGGCTAAACTA	
BBX28^{C47,50A}-F	GTCTTCTCGCCAGCGCTGCCCAGTCTC	
BBX28^{C47,50A}-R	GAGACTGGGCAGCGCTGGCGAGAAGAC	
BBX28^{C70,73A}-F	CTCCGTCGCCGAGTCAGCCGTCGCTC	
BBX28^{C70,73A}-R	GAGCGACGGCTGACTCGGCGACGGAG	
